# Effects of insect diets on the gastrointestinal tract health and growth performance of Siberian sturgeon (*Acipenser baerii* Brandt, 1869)

**DOI:** 10.1186/s12917-019-2070-y

**Published:** 2019-10-17

**Authors:** Agata Józefiak, Silvia Nogales-Mérida, Mateusz Rawski, Bartosz Kierończyk, Jan Mazurkiewicz

**Affiliations:** 1 Department of Preclinical Sciences and Infectious Diseases, Poznan University of Life Sciences, Wołyńska, 35, 60-637 Poznań, Poland; 2 Hipromine. S.A., Robakowo, Poland; 3 Division of Inland Fisheries and Aquaculture, Institute of Zoology, Poznan University of Life Sciences, Wojska Polskiego 71c, 60-625 Poznań, Poland; 4 Department of Animal Nutrition, Poznan University of Life Sciences, Wołyńska 33, 60-637 Poznań, Poland

**Keywords:** Siberian sturgeon, Insect meals, Fish nutrition, Microbiology, *Tenebrio molitor*, *Hermetia illucens*

## Abstract

**Background:**

Insects in the fish diet are a natural source of protein, fat, and other nutrients. These meals are considered an ecological replacement for fishmeal to improve growth parameters. The application of insect meals to fish diets has been studied, especially in continental fish. Data regarding the effects of insect meals on the gut health of Siberian sturgeon are not available. This study investigated the effects of full-fat *Hermetia illucens* (HI) and *Tenebrio molitor* (TM) meals on the gut health of juvenile Siberian sturgeon. Growth performance, gastrointestinal tract (GIT) histomorphology and the microbiome composition of juvenile Siberian sturgeon were analyzed.

**Results:**

The inclusion of insect meals did not affect the growth performance or the survival rate. In the gastrointestinal tract histomorphology, a reduction in the mucosa thickness with the HI treatment was observed. In contrast, fish fed the TM diet had an increase in the thickness of the muscular layer. There were no observed significant differences in villus height among treatments. The analysis of the selected microbiota populations in the Siberian sturgeon gastrointestinal tract showed that insect addition affected the composition of the microbiome. The greatest effect on bacterial populations (*Clostridium leptum* subgroup*,* Enterobacteriaceae, *Clostridium coccoides – Eubacterium rectale* cluster, *Aeromonas* spp., *Bacillus* spp., *Carnobacterium* spp., *Enterococcus* spp. and *Lactobacillus* group) was observed with the HI diet (*P* < 0.05). The TM-based diet increased counts in the following bacterial groups: *Clostridium coccoides – Eubacterium rectale* cluster, *Bacillus* spp., *Carnobacterium* spp., and *Enterococcus* spp. In contrast, the TM diet decreased the total number of bacteria*.* The TM diet did not significantly affect the *Clostridium leptum* subgroup*,* Enterobacteriaceae, *Aeromonas* spp. or the *Lactobacillus* group.

**Conclusions:**

Fish meal replacement by the inclusion of 15% of full-fat *Hermetia illucens* and *Tenebrio molitor* (15%) meals did not affect the growth performance, survival rate or villus height of juvenile Siberian sturgeon. The present study suggests that an *H. illucens-*based diet positively affects the gut microbiota composition and intestinal morphology of juvenile Siberian sturgeon without negative changes in the villus height.

## Background

The Siberian sturgeon (*Acipenser baerii,* Brandt, 1869) is one of the most valuable fish species in aquaculture. It is farmed for the production of caviar and high meat quality for human consumption [9, 10]. This species, as well as others in the family Acipenseridae, are known to eat insects, mainly chironomids (Chironomidae) and mayflies (Ephemeroptera) [25, 28, 45], in the wild. The primary prey of shovelnose sturgeon (*Scaphirhynchus platorynchus*) and pallid sturgeon (*S. albus*) are the aquatic larvae of Trichoptera, Ephemeroptera, and Diptera [31]. Insects in the fish diet are a source of protein, lipids, and bioactive compounds, including vitamins, chitin, and antimicrobial peptides (AMPs), which are known to improve the animal immune system and can modulate the microbiota composition of the gastrointestinal tract [38–41, 61]. In addition, certain insect meals can also provide minerals such as calcium, as with *H. illucens,* or phosphorus, as with *Musca domestica* [51], although the contents of these nutrients depend on the insect diet [30].

Although juveniles consume insects in natural conditions, in aquaculture, their diets contain fishmeal (FM) as the main source of protein [15, 56]. However, the use of FM is an environmental concern due to the high amount of wild fish captured for FM production [34]. Many alternative protein sources have been studied for general fish nutrition to replace fishmeal. The most available are plant meals. However, some plant meals resulted in growth depression, i.e., soybean meal (SBM) [43], soy protein concentrate, and rapeseed meal [55]. Other meals, such as sesame oil cake and corn gluten [37], caused a reduction in growth performance in cases with inclusion levels higher than 24% and was the same with wheat gluten [4]. This may be partially explained by the presence of antinutritional factors such as phytic acid, lectins, saponins, protease inhibitors, glucosinolates, and tannins [18]. Additionally, to achieve optimal growth performance using plant meals, it is necessary to use essential amino acid supplementation [86]. Thus, the use of plant meals is considered a cause of health problems in sturgeon [46, 47].

Recently, insect meals have been promoted to replace FM and/or SBM for fish nutrition, such as the larvae of green bottle fly (*Lucilia sericata*) used in gilthead seabream (*Sparus aurata*) meal [11] or superworm (*Zophobas morio*) for Nile tilapia, *Oreochromis niloticus* [36]. One of the most studied insects as an additive for fish nutrition is the mealworm (*Tenebrio molitor*), which has been evaluated in rainbow trout (*Oncorhynchus mykiss*) [5], catfish (*Clarias gariepinus*) [60], common catfish (*Ameiurus melas*) [73], guppies (*Poecilia reticulata*) [2], and European seabass (*Dicentrarchus labrax*) [21]. The second is the black soldier fly (*Hermetia illucens*), which has also been evaluated in rainbow trout (*Oncorhynchus mykiss*) [14, 70, 80], Nile tilapia [58], common carp (*Cyprinus carpio*) [48] and European seabass [50]. However, in the available literature, there is no information about the application of mealworm and/or black soldier fly meals for sturgeon nutrition.

Some of the most important factors of animal health include growth performance parameters and gastrointestinal tract (GIT) conditions. There are many data on the impact of nutrition on the gastrointestinal microbiota and gut health [6–8, 11, 16, 20, 26, 30, 40, 72]. Intestinal diseases can develop as an effect of unbalanced diets resulting in disturbances in the homeostasis of the GIT microbiome.

Therefore, the current study was designed to evaluate the effects of partial fishmeal replacement with full-fat mealworm and black soldier fly meals for Siberian sturgeon with regard to fish performance parameters, survival rate, GIT microbiota composition and gut histomorphology.

## Results

### Growth and feed efficiency

There were no significant differences among the treatments with regard to BWG, SGR, FCR, and PER at the end of the experimental period (*P* > 0.05). The survival rate at the end of the trial was 100% for all the treatments (Table 1).

**Table 1:** Growth and feed utilization parameters of Siberian sturgeon (*Acipenser baerii* Brandt) fed three experimental diets in which fishmeal was partially replaced with full-fat *Hermetia illucens* meal (15%) and *Tenebrio molitor* meal (15%) for 60 days. Data are presented as the mean ± standard error

	Diets			SEM	*p-value*
	CT	TM	HI		
IBW (g)	636.67	648.33	646.67	6.45	0.4363
FBW (g)	1196.81	1195.04	1194.81	16.44	0.1178
BWG (g)	546.67	553.33	555.00	7.7700	0.9217
FCR^a^	1.47	1.47	1.48	0.03	0.9356
SGR^b^	1.03	1.03	1.03	0.02	0.9515
PER^c^	1.52	1.50	1.52	0.0100	0.8907

All values are means of triplicate cases (n = 3). Different alphabetic superscripts in the same row indicate significant differences at α < 0.05

*IBW* initial body weight, *FBW* final body weight, *BWG* body weight gain

^a^Feed conversion ratio, FCR = feed offered (g)/biomass gain (g)

^b^Specific growth rate (% day^−1^), SGR = 100 x ln (final weight/initial weight)/days.

^c^Protein efficiency ratio, PER = biomass gain (g)/protein offered (g)

### Histomorphology

The mucosa thickness and muscular layer, but not villus height, (Fig. 2) were affected by dietary treatments (*P* < 0.05). The HI diet caused the highest reduction in mucosal thickness (118 μm) compared to that of the fish fed the control diet (154 μm) and the fish fed the *T. molitor* (145 μm) diet (*P* < 0.05)*.* In contrast, the *T. molitor* diet increased the muscular layer thickness (300 μm) in comparison to that in the fish fed the HI diet (262 μm) and the control diet (243 μm) (*P* < 0.05).

### Microbiota analysis in the gastrointestinal tract (GIT)

The gut microbiota analysis of juvenile Siberian sturgeon fed with HI and TM revealed statistically significant (*S <* 0.05) changes in the following taxonomic groups: the *Clostridium leptum* subgroup*,* Enterobacteriaceae, *Clostridium coccoides – Eubacterium rectale* cluster, *Aeromonas* spp., *Bacillus* spp., *Carnobacterium* spp., *Enterococcus* spp. and the *Lactobacillus* group (Table 2). The HI-based diet group had the highest total number of bacteria in GIT digesta. Compared to that of the CT group, the HI group had significantly increased bacterial counts observed for all analyzed groups of GIT microbiota, including the *Clostridium leptum* subgroup, Enterobacteriaceae, *Clostridium coccoides – Eubacterium rectale* cluster, *Aeromonas* spp., *Bacillus* spp., *Carnobacterium* spp., *Enterococcus* spp. and the *Lactobacillus* group, which changed significantly according to feed type (*P <* 0.05) (Table 2). The TM-based diet increased counts in the following groups of bacteria: *Clostridium coccoides – Eubacterium rectale* cluster, *Bacillus* spp., *Carnobacterium* spp., and *Enterococcus* spp. In contrast, the TM diet decreased the total number of bacteria*.* No statistical effect of the TM diet compared to the CT diet was observed in the case of the *Clostridium leptum* subgroup*,* Enterobacteriaceae, *Aeromonas* spp. and the *Lactobacillus* group.

**Table 2:** Selected intestinal microbiota populations of Siberian sturgeon digesta at the end of the experimental period

Target	Diets			SEM	*p-value*
	CT	HI	TM		
LOG CFU/g of digesta					
Total number of bacteria	8.27^b^	8.87^a^	8.14^c^	0.0479	0.0001
*Clostridium leptum subgroup*	7.66^b^	8.12^a^	7.73^b^	0.0349	0.0001
Enterobacteriaceae	7.69^b^	7.94^a^	7.73^b^	0.0301	0.0004
*Clostridium coccoides – Eubacterium rectale* cluster	7.40^b^	7.66^a^	7.55^a^	0.0287	0.0008
*Aeromonas* spp.	8.10^b^	8.45^a^	8.13^b^	0.03	<.0001
*Bacillus* spp.	8.02^c^	8.42^a^	8.25^b^	0.03	<.0001
*Carnobacterium* spp.	7.97^c^	8.34^a^	8.20^b^	0.03	<.0001
*Enterococcus* spp.	7.81^c^	8.21^a^	8.03^b^	0.03	<.0001
*Lactobacillus* group	7.39^b^	8.19^a^	7.96^ab^	0.12	0.0205

All values are means of nine cases (*n* = 9). Different letters indicate significant differences between treatments (*P* < 0.05)

## Discussion

To the authors’ knowledge, the present study is the first attempt to replace fishmeal with full-fat *H. illucens* and *T. molitor* meals for Siberian sturgeon nutrition. Currently, the main source of protein for farmed sturgeon is fishmeal with partial inclusion of plant meals, especially soybean meal [12, 35, 74, 83]. However, the present results suggest that insect meals can also substitute for FM in Siberian sturgeon diets without negative effects on growth performance. Moreover, considering the 15% inclusion rate, *H. illucens* meal represented a 30% replacement of FM protein in the diet, and in the case of *T. molitor*, a 40% replacement. Similar values have been obtained in experiments conducted with *O. mykiss* [5], *C. gariepinus* [60] and *C. carpio* [48], which also consume insects as part of their natural diet.

It is well documented that dietary factors may affect the intestinal morphology, even changing the length of the gut as well as the internal structures, such as the elongation or shortening of the villi, the modification of the goblet cells [76], and the migration of inflammatory cells [83]. Consequently, this affects animal gut health [57]. This could be the case in the muscularis layer when it thickens, which has been associated with the improvement of digestion and absorption [77]. The opposite effect is associated with a decrease in motility in the digestive tract as a degenerative process in aging [78].

Therefore, in the current experiment, modification of the GIT structures due to the inclusion of full-fat insect meals in the Siberian sturgeon diet could be beneficial to the animals by improving the function of the GIT. Interestingly, changes in GIT structures, particularly mucosal and muscular layer thickening, indicate the adaption of the GIT to insect-based diets, which are much closer to natural conditions [28].

The structure of the digestive tract differs according to fish species. The differences develop in the embryonic stage and are the first factor in gastrointestinal bacterial community formation. In general, *Aeromonas*, *Pseudomonas*, *Clostridium* and *Bacteroides* predominate in fish GITs. However, *Bacteroides* appears late in the gastrointestinal tract of fish, as late as on the 44th day after hatching, but they become predominant in the intestines of adult fish.

In fish as well as in other vertebrates, gastrointestinal microbiota populations strictly correlate with diet and GIT functions, as well as the health status and performance of the animals. *H. illucens* and *T. molitor* are rich sources of small proteins and peptides with balancing effects on GIT microbiota [39]. They are known to act as prebiotics, improving the gut absorptive area [27], selectively stimulating the growth of beneficial bacteria over detrimental pathogenic bacteria [27], fungi and viruses [85]. They also improve the oxidative status of fish [27], promoting the colonization of beneficial gut microbiota. In addition, chitin in the exoskeleton of insects has antioxidant activity in low amounts as well as antifungal and antiviral properties [3]. Additionally, GIT morphology and animal growth are dependent on the gut bacterial community [66].

All of the analyzed bacterial groups consist of important bacterial populations of the GIT microbiome. Moreover, *Lactobacillus, Enterococcus, Carnobacterium, Bacillus, Aeromonas* and *Clostridium* are some of the most common probiotics used in aquaculture practices, which suggests that insect meals with the above bacterial strains may provide health benefits for Siberian sturgeon breeding [59].

Phylogenetic analyses of the 16S rRNA genes from the Siberian sturgeon gut microbial communities showed that the most abundant phyla detected in the fish hindguts were Fusobacteria, Firmicutes and Proteobacteria [22, 23]. Fusobacteria are represented mainly by *Fusobacterium* [22, 23]. The most frequent classes within the Firmicutes phylum were Clostridia and Bacilli [22, 23]. The most dominant bacterial populations in the Proteobacteria gamma subclass was the genus *Aeromonas* and the family Enterobacteriaceae [23, 32]. *Aeromonas* are frequently identified in carnivores, omnivores and planktivores and make up natural and important parts of the fish gut microbial community [13]. The genus *Aeromonas* cover a diverse group of gram-negative bacteria that are commonly present in aquatic environments and soil, as well as in various animals and humans. Aeromonads are ubiquitous bacteria in fresh water and have been identified as potential pathogenic bacteria that cause fish skin changes and a variety of human diseases associated with intestinal and extraintestinal infections [65]. *Aeromonas* is also a potential foodborne pathogen for humans. However, the epidemiology of human disease is still unclear; therefore, they need to be considered with regard to public health [62]. A study performed on Arctic charr, *Salvelinus alpinus,* showed that a commercial diet changed gut microbial diversity [71, 72]. In the GIT of fish maintained in freshwater and seawater, approximately 55% of the bacterial flora was Enterobacteriaceae. However, when the fish were fed a commercial diet, the dominant microflora in feces were *Aeromonas* and *Pseudomonas* [72]. According to the results of other studies, *Aeromonas* and *Lactobacillus* bacteria predominate in the intestinal microbiome of fish inhabiting natural water bodies, whereas Enterobacteriaceae, which comprise up to 50% of all bacteria, are prevalent in the microbiota of farmed fish that are fed artificial food [20]. However, modification of the bacterial community is an important factor for disease prevention and treatment.

In the current study, *Aeromonas* spp. increased with the HI treatment, while a TM diet did not statistically change the aeromonad population, which may suggest that HI (15%) may change the GIT microbial diversity of juvenile Siberian sturgeon, leading to aeromonad domination in relation to Enterobacteriaceae.

High values of the Enterobacteriaceae family were also observed in the digesta of fish fed the HI diet. This could be explained according to the findings of Borrelli et al. [8], who noted that *H. illucens* significantly increased the diversity within the microbial population. In addition, the presence of bacteria that belong to the Enterobacteriaceae family is considered normal in fish farmed near human populations [80–82].

*Clostridium leptum* subgroups are known to produce butyrate products, which contribute to human intestinal health [44, 52]. Changes in the *Clostridium leptum* subgroup is an indicator of the health status of the gut. Species of this genus produce organic acids, including butyrate, acetate, lactate, or formate, but not propionic and succinic acids, as primary products of dietary fiber fermentation. Reduction in the number and diversity of *the Clostridium leptum* subgroup is observed in human Crohn’s disease and ulcerative colitis [44], which could contribute to reduced short-chain fatty acids. The abundance of *E. rectale* and *Clostridium leptum* indirectly affects epithelial cell structure and function, particularly in the lower regions of the GIT [52]. In our study, the HI additive significantly increased the counts of *Clostridium leptum,* while no significant differences were observed in the TM diet group and the control group. Increased *Clostridium* counts were also observed in the *Clostridium coccoides – Eubacterium rectale* cluster in the HI and TM diets.

Another potentially positive effect on gut health status is an increase in the number of *Bacillus* spp*.*, which one of the most common probiotics used in aquaculture [23]. A study performed by Ghosh et al. (2002) identified different members of the *Bacillus* genus as *B*. *circulans*, *B. pumilus* and *B. cereus* [24]. The study of bacterial isolates indicated that they could adapt to a wide range of temperatures and pH. Autochthonous bacteria that remained present in the GIT possessed enzymatic activity that might have had beneficial effects on fish health [24]. The effect of endogenous *Bacillus circulans* was also investigated by Geraylou et al. [23] in a study performed on juvenile Siberian sturgeon (*Acipenser baerii*). The authors identified *Lactococcus lactis* subsp. *lactis* and *Bacillus circulans* isolated from the hindgut of juvenile Siberian sturgeon as a new class of candidate prebiotics [23]. Moreover, several studies have demonstrated the immunomodulatory effects of *Bacillus* spp., including the improvement of phagocytosis in *C. catla* and the resistance to salinity, temperature, ammonia and pH changes by the larvae of *Acipenser percicus* [17, 23]*.* In the current study, *Bacillus* spp. were increased with HI and TM treatments, which may suggest improvement of the positive bacterial barrier against fish pathogens and may enhance host defense immunomodulatory effects.

Another bacterial group isolated from the gastrointestinal tract that produces inhibitory substances against bacterial fish pathogens was *Carnobacterium* spp. Lactic acid bacteria (LAB) were isolated from fish and screened for bacteriocin production and immunomodulatory effects [64]. The study by Pilet et al. (1995) indicated that *Carnobacterium piscicola* and *Carnobacterium divergens* isolated from fish could produce bacteriocins active against *Listeria monocytogenes* [64]. Bacteriocin-producing bacterial strains belonging to the genera *Carnobacterium* and *Lactococcus* and *Enterococcus* were isolated from fish intestines, smoked fish and even marine fish*. The genus Enterococcus includes more than 30 species. However, the most prevalent species in foods are Enterococcus faecalis and Enterococcus faecium. Some enterococci have bacteriocinogenic potential and are capable of inhibiting the growth of pathogenic bacteria.*

In our study, the bacterial counts of *Carnobacterium* spp., *Enterococcus* spp. and the *Lactobacillus* group were significantly different. The results of the TM treatment on LAB bacteria indicated an increase in *Carnobacterium* spp. and *Enterococcus* spp., while no differences were indicated in the *Lactobacillus* group of bacteria. This may suggest a greater increase in LAB in the HI diet than that in the TM diet. However, both HI and TM diets caused an increase in probiotic bacterial populations to have a healthy effect on juvenile Siberian sturgeon.

Another possible cause could be attributed to dietary fat and carbohydrates, which are considered major factors for modification of the gut microbial community profile. Many studies reported that a high-fat diet increased the population of Enterobacteriaceae and *Lactobacillus* spp. [29, 49]. These results were also confirmed in our studies. The diet with HI had a higher fat content with greater Enterobacteriaceae and *Lactobacillus* group populations than did the TM diet, which did not cause changes in the Enterobacteriaceae and *Lactobacillus* bacterial communities. The effects of different lipid and carbohydrate contents in the diets were observed by Ringø and Olsen [71] in *Salvelinus alpinus* fed with different levels of carbohydrates/lipids. Nevertheless, as Borrelli et al. (2017) noted, the acquired microbial richness may potentially provide further metabolic capabilities to the host, which could be the case for Siberian sturgeon fed a diet with HI [8]. In the case of the TM diet, the effects of this insect meal seemed to be similar to those of the CT diet. However, if we examine growth performance and microbiota, these values were not enough to cause health problems for Siberian sturgeon or altered weight gain during the experimental period.

In general, the increase of bacterial populations observed in this study should be considered a positive effect that may improve fish health. Intestinal microbiota play an indispensable role in the origin of gut health. Host microbiota homeostasis is an important factor in the first line of defense against pathogens. Moreover, diversification of the microbial population of the fish GIT is very important in establishing health status, and it can diversify enzymatic potential to interfere with host metabolism [20]. Gastrointestinal bacteria take part in nutrient digestion to provide the host enzymes, amino acids and vitamins [49]. Furthermore, the diversity of symbiotic gut microflora might also be a potential mechanism for the regulation of bacterial homeostasis by IgA. IgA is the major antigen-specific mechanism by which the immune system directly interacts with and influences the luminal microbiota and supports host–microbiota homeostasis [63].

Additionally, the insect meals seemed to improve the relationship between GIT morphology and the gut microbiome, promoting the health of the fish with survival and growth performance similar to those fed with fishmeal. That was not the case in *S. aurata* fed with a mix of plant meal, which seriously affected the survival of the animals; Estruch et al. (2015) associated this mortality with certain bacterial consortia within the plant meal diet [16].

## Conclusion

The current preliminary study demonstrated that the inclusion of 15% of *H. illucens* and *T. molitor* full-fat meals in Siberian sturgeon diets resulted in the same growth performance and feed efficiency as those obtained with fishmeal. The HI-based diet caused certain modifications of the intestinal histomorphology and microbiology, namely, a reduced thickness of mucosa, increased muscle layer thickness and an increase in the total number of bacteria in the following groups: the *Clostridium leptum* subgroup*,* Enterobacteriaceae, *Clostridium coccoides – Eubacterium rectale* cluster, *Aeromonas* spp., *Bacillus* spp., *Carnobacterium* spp., *Enterococcus* spp. and the *Lactobacillus* group.

In contrast, the TM diet increased the thickness of the muscular layer. Both the HI and TM diets did not elicit significant differences in villus height. There was no effect of the TM diet on the intestinal population of the *Clostridium leptum* subgroup*,* Enterobacteriaceae, *Aeromonas* spp., the *Lactobacillus* group, and *Enterococcus* spp. Changes in microbiota composition and intestinal morphology contributed to the gut health status of Siberian sturgeon. Moreover, it could be concluded that the *H. illucens*-based diet has a higher potential for modulation of the GIT microecosystem compared to the *T. molitor* diet*.* However, these changes could be attributed to other factors that did not affect the performance of the fish during the experiment and need further microbiological studies. In addition, other trials should be conducted in Siberian sturgeon fed different levels of insect meals to find the maximum amount that the animal could accept without negatively affecting the feed efficiency parameters and health. In addition, we should determine whether the morphological changes would be harmful to animal health or only an adaptation to the insect meal.

The insect diet including *Hermetia illucens* (15%) and *Tenebrio molitor* (15%) did not affect the growth performance, survival rate or villus height and can be considered as a partial replacement of other protein sources in juvenile Siberian sturgeon. The results of the present study indicated that an insect diet including *H. illucens* can have a positive effect on the gut microbiome composition and intestinal morphology of juvenile Siberian sturgeon.

## Methods

### Experimental diets

Three extruded isonitrogenous (53.20 ± 0.12% CP) and isoenergetic (17.50 ± 0.07 MJ) diets were prepared by replacing fishmeal (FM) in the control (CT) diet with 15% *Hermetia illucens* (HI) larvae meal and 15% *Tenebrio molitor* (TM) larvae meal (Table 3). These three diets were referred to as the CT diet, HI diet, and TM diet. The full-fat insect meals were prepared by HiProMine S.A. (Poland). They were dried at 50 °C for 48 h until they reached 95% dry matter.

**Table 3:** Ingredients and composition (%) of experimental diets for Siberian sturgeon

Ingredient	Diets		
	CT	HI	TM
Fishmeal^a^	26	18.4	15.6
Mealworm, TM (*Tenebrio molitior*)^b^	0	0	15
Black soldier fly, HI (*Hermetia illucens*)^b^	0	15	0
Red blood cells (dried)^c^	10	10	10
Yeasts^d^	8	8	8
Post-extraction soybean meal^e^	10	10	10
Dried whey	5	5	5
Wheat gluten	7	7	7
Wheat meal	19.1	15.9	17.1
Fixogran	2	2	2
Rapeseed oil	10.1	5.9	7.5
Lecithin	0.5	0.5	0.5
Premix^f^	1.5	1.5	1.5
Vitazol AD_3_E^g^	0.1	0.1	0.1
Choline chloride	0.2	0.2	0.2
Vitamin C	0.5	0.5	0.5
TOTAL %	100	100	100

CT – control treatment – 26% of fishmeal, HI – 15% of *Hermetia illucens* full-fat meal and 18.4% of fishmeal, TM – 15% of *Tenebrio molitor* full-fat meal and 15.6% of fishmeal

^a^Danish fishmeal: DM: 90%; CP: 72%; CF: 12%; Ash: 14%. Skagen, Denmark

^b^ Black soldier fly meal: DM: 97.5%; CP: 40.4%; CL: 33.5%; Ash: 7.13%. Mealworm meal: DM: 93.9%; CP: 56.3%; CL: 25.3%; Ash: 4.53%. HiProMine S.A. Poland

^c^Spray dried, 900 g kg^− 1^ protein, APC Europe, Spain

^d^Yeast, 45% protein, 6% ash

^e^Solvent extracted: 45% protein

^f^Polfamix W, BASF Polska Ltd. Kutno, Poland – containing per 1 kg: vitamin A 1000000 IU, vitamin D3 200,000 IU, vitamin E 1.5 g, vitamin K 0.2 g, vitamin B1 0.05 g, vitamin B2 0.4 g, vitaminB12 0.001 g, nicotinic acid 2.5 g, D-calcium pantothenate 1.0 g, choline chloride 7.5 g, folic acid 0.1 g, methionine 150.0 g, lysine 150.0 g, Fe 2.5 g, Mn 6.5 g, Cu 0.8 g, Co 0.04 g, Zn 4.0 g, J 0.008 g,

^g^Vitamin premix AD_3_EC, BIOWET Drwalew, Poland – contains in 1 kg: vitamin A 50000 IU, vitamin D_3_ 5000 IU, vitamin E 30.0 mg, vitamin C 100.0 mg

In all diets, blood meal, yeasts, postextraction soybean meal, dried whey and wheat gluten were added as protein sources supplements at the same levels. The uses of these meals are approved according to EU regulations.

The diets were prepared with a cooking extrusion process using a semi-industrial single-screw extruder (type S-60, METALCHEM Gliwice, Poland). The extrusion conditions were as follows: 90 °C cylinder temperature in the zone of increasing pressure, 100 °C cylinder temperature in the zone of high pressure, 110 °C head temperature, 52 rpm speed screw and 6 mm nozzle diameter.

### Proximate composition and amino acid analysis

Chemical analyses of the dietary ingredients were performed prior to diet formulation. Diets and their ingredients, as well as the whole fish, were analyzed according to AOAC [1] procedures. Crude protein, (N × 6.25) was analyzed by the Kjeldahl method after acid digestion (Kjeltec 2300 Auto Analyser, Tecator Hogänas, Sweden). Crude lipid (CL) was extracted with methyl–ether (Soxtec 1043 extraction unit, Tecator, Sweden). Dry matter analysis was performed using the Polish Standard PN-ISO 6496 and ash analysis was performed using the Polish Standard PN–76 R 64795. For crude fiber determination, the Polish Standard PN-EN ISO 6865 was used (Table 4). All analyses were performed in triplicate.

**Table 4:** Chemical composition of the experimental diets, including amino acids expressed in dry matter

Parameter	Diets		
	CT	HI	TM
Dry matter (%)	90.08	90.41	90.42
Crude protein (%)	53.02	53.21	53.36
Crude fat (%)	19.67	19.85	19.28
Crude fibre (%)	2.81	2.79	2.53
Ash (%)	7.49	7.51	7.48
Nitrogen-free-extract (%)	7.09	7.05	7.77
Energy (MJ/kg)	17.48	17.57	17.50
*Indispensable Amino Acids (g/100 g)*			
Arginine	2.68	2.71	2.51
Histidine	1.71	1.76	1.60
Isoleucine	1.55	1.57	1.46
Leucine	4.53	4.59	4.24
Lysine	3.72	3.76	3.49
Methionine	1.38	1.40	1.28
Phenylalanine	2.52	2.57	2.38
Serine	1.45	1.47	1.37
Threonine	2.88	2.93	2.74
*Dispensable Amino Acids (g/100 g)*			
Alanine	3.10	3.13	2.91
Aspartic acid	4.56	4.63	4.30
Cysteine	0.38	0.37	0.38
Glutamic acid	8.51	8.65	8.00
Glycine	2.73	2.78	2.52
Proline	2.88	2.94	2.65
Tyrosine	2.23	2.29	2.11
*IAA/DAA*^a^	*0.85*	*0.85*	*0.86*

^a^*IAA* Indispensable Amino Acids, *DAA* Dispensable Amino Acids

The amino acids of the experimental diets were analyzed by a certificated company, J.S. Hamilton S.A., Poland, where 17 amino acids were detected. The composition analysis of the experimental diets (Table 4) showed that the inclusion of insect meal did not affect the amino acid values or the relationship of essential and nonessential amino acids (EAA/NEAA), which were between 0.85 and 0.86.

### Growth trial and fish sampling

A total of 180 juvenile Siberian sturgeon (640 g ± 3.9) were used in the trial. Twenty fish were randomly distributed per tank. All animals were brought to Feed Production Technology and Aquaculture Experimental Station in Muchocin of Poznań Univeristy of Life Sciences, from Acipenseridae Fish Breeding Station of the Stanisław Sakowicz Inland Fisheries Institute in Olsztyn, Poland. Prior to the feeding trial, all fish were acclimated to the experimental rearing conditions for 1 month and fed the commercial diet “Aller Silver” (45% crude protein, CP; 15% crude lipid, CL, Aller Aqua Poland). The fish were housed in nine fiberglass tanks (600 dm^3^ capacity). Each dietary treatment was performed in 3 replications, using 20 fish in each.

**Fig. 1:**
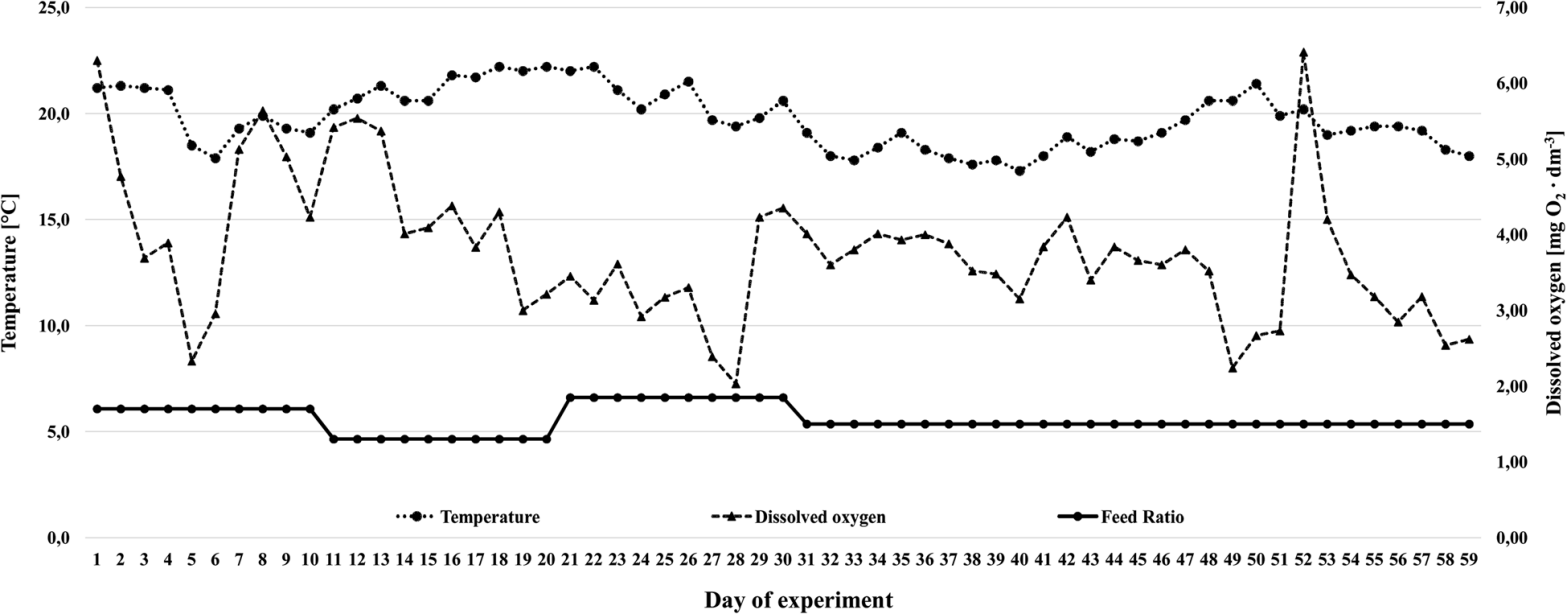
Relation between temperature, dissolved oxygen, and feed ratio

The experimental unit was arranged as an open-flow system; the water came directly from a river and passed through a mechanical prefiltration chamber before it entered the tanks. A water heating system was not used in this experiment to best replicate natural water temperature and the dissolved oxygen variation, which are present in European aquaculture, to simulate commercial farm conditions. The water flow allowed the complete replacement of the total tank volume once per hour. During the growth tests, the mean water temperature was 19.8 ± 1.4 °C, the dissolved oxygen (Fig. 1) was 3.8 ± 0.9 mg O_2_ dm^− 3^ (WTW Multi Line P4 with optical oxygen sensor FDO 924, WTW, Weilheim, Germany), and the water pH ranged from 6.9 to 7.2 (WTW Multi Line P3 pH meter, WTW, Weilheim, Germany).

**Fig. 2:**
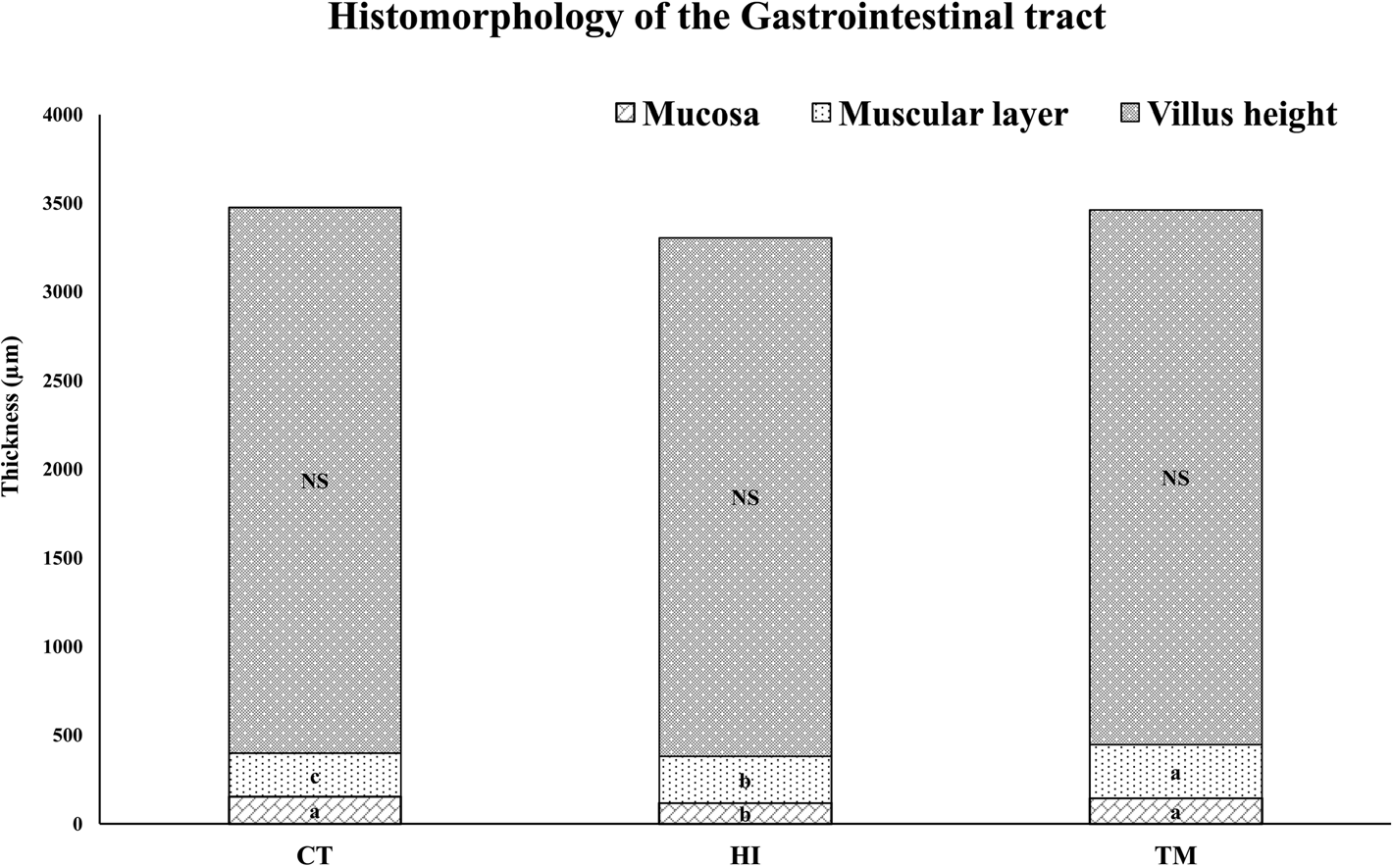
Histomorphology of the proximal intestine portion of Siberian sturgeon fed with the experimental diets at the end of the 60 days (*n* = 12). Different letters indicate significant differences between treatments (*P* < 0.05)

Fish were fed every day with an automatic band feeder for 12 h per day (08:00 a.m. to 08:00 p.m.). The daily feed rations varied from 1.8 to 1.4% according to the feeding rate as outlined by Hung, Lutes, Shqueir, and Conte [33], the biomass weight and the water temperature. The sizes of rations were adjusted every 10 days based on weight monitoring and feed intake observations (Fig. 2). The trial lasted 60 days. During this time, the daylight length was from 16:55 to 13:21 h.

All fish were weighed at 10-day intervals according to Mazurkiewicz et al. (2010) [54]. Prior to weighing, the fish were anesthetized by immersion in 130 mg/L tricaine methanesulfonate (MS–222, Sigma Aldrich) solution. At the end of the growth trial, all fish were individually weighed. Three animals per tank were euthanized by immersion in 500 mg/L of MS-222 solution for dissection (*n* = 9/treatment, *n* = 27 in total), all remaining animals were further maintained in the experimental station. Three intestinal tissue samples per tank were collected for histological analysis as well as for the gut microbial analysis (n = 9/treatment, n = 27 in total). During the experimental period, the following parameters were considered: mean body weight gain of individual fish (WG) expressed in grams, feed conversion ratio (FCR), specific growth rate (SGR % day^− 1^) and protein efficiency ratio (PER), which were calculated according to survival rates (*n* = 3/treatment, *n* = 9 in total).

### Gastrointestinal histology

Proximal intestine tissue samples (n = 9/treatment, *n* = 27 in total) were fixed in a freshly prepared formaldehyde solution (40 g/L of formaldehyde prepared in 0.01 M PBS, pH = 7.4) immediately after dissection and incubated for 12 h. Afterward, the samples were processed and analyzed using the methodology described in details in Rawski et al. 2016 [68]. The stained samples were visualized with Aziophot Opton light microscope at 40x magnification. The following parameters were measured: mucosal thickness, muscular layer thickness and villus height. The length of the 10 villi with complete structures were measured in 12 serial slides using a glass master micrometer (0.01 mm, PZO, Warsaw, Poland) and analyzed as the means.

### Microbial community analysis by fluorescent in situ hybridization (FISH)

The samples of gastrointestinal content collected during fish dissection were immediately frozen and stored at − 80 °C (n = 9/treatment, n = 27 in total). The FISH and visualization procedures were performed according to Rawski et al. 2016 [68]. The oligonucleotide probes used for this study (Table 5) were selected from the literature [19, 53] and have been used successfully in our previous studies on poultry and turtles [38, 42, 67–69]. The numbers of detected bacteria are expressed in colony-forming units/g of digesta (CFU/ml) and were calculated according to the following equation:

**Table 5:** Oligonucleotide probes used in fluorescent in Situ hybridization

Target	Probe	Sequence (5′ to 3′)	References
*Enterobacteriaceae*	Enter1432	CTTTTGCAACCCACT	Sghir et al., (2000) [79]
*Clostridium leptum* subgroup	Clept1240	GTTTTRTCAACGGCAGTC	
*Clostridium coccoides* —*Eubacterium rectale cluster*	Erec482	GCTTCTTAGTCARGTACCG	Franks et al., (1998) [19]
*Aeromonas* spp.	Aer66	CTA CTT TCC CGC TGC CGC	Huber et al., (2004) [32]
*Bacillus* spp.	Bmy843	CTT CAG CAC TCA GGT TCG	Salzman et al., (2002)
*Carnobacterium* spp.	CAR193	AGC CAC CTT TCC TTC AAG	Huber et al., (2004) [32]
*Enterococcus* spp.	Enfm93	CCG GAA AAA GAG GAG TGG C	Waar et al., (2005)
*Lactobacillus* group	Lab722	YCA CCG CTA CAC ATG RAG TTC CAC T	Sghir et al., (1998)

$$ \log\;CFU/g=\log \left(N\times \left(\frac{WA}{PA}\right)\times \left(\frac{Sweight+ Dweight}{Sweight}\right)\times \left(\frac{1000}{Svolume}\right)\right) $$

where N = the number of visible bacterial cells, WA = the work area of the filter, PA = the picture area, S_weight_ = the sample weight, D_weight_ = the diluting factor weight, and S_volume_ = the volume of the sample pipetted onto the filter.

### Statistical analysis

All data were tested for normal distribution using the Kolmogorov-Smirnov test. The analysis of variance (ANOVA) was performed. The significance of differences among the groups was determined by Duncan’s multiple range test at the significance level of *P* < 0.05. The following general model was used:

$$ {Y}_i=\mu +{\alpha}_i+{\delta}_{ij} $$

where Y_i_ is the observed dependent variable, μ is the overall mean, α_i_ is the effect of the diet, and δ_ij_ is the random error.

## Data Availability

All data are included in this published article. The raw datasets are available from the corresponding author on reasonable request.

## Abbreviations

AMP: Antimicrobial peptide
AOAC: The Association of Analytical Communities
BWG: Body weight gain
CL: Crude lipid
CT: Control
EAA: Essential amino acids
FCR: Feed conversion ratio
FISH: Fluorescent in situ hybridization
FM: Fishmeal
GIT: Gastrointestinal tract
HI: *Hermetia illucens*
LAB: Lactic acid bacteria
MS–222: Tricaine methanesulfonate
NEAA: Nonessential amino acids
PER: Protein efficiency ratio
SBM: Soybean meal
SGR: Specific growth rate
TM: *Tenebrio molitor*
